# Optimizing the Bioprocesses of Bacteriocin Production in *Lacticaseibacillus paracasei* HD1.7 by the “Acetate Switch”: Novel Insights into the Labor Division Between Energy Metabolism, Quorum Sensing, and Acetate

**DOI:** 10.3390/foods14152691

**Published:** 2025-07-30

**Authors:** Weige Yao, Rui Sun, Wen Zhang, Jie Kang, Zhenchao Wu, Liangyang Mao, Ying Yang, Shuo Li, Gang Song, Jingping Ge, Wenxiang Ping

**Affiliations:** Engineering Research Center of Agricultural Microbiology Technology, Ministry of Education & Heilongjiang Provincial Key Laboratory of Plant Genetic Engineering and Biological Fermentation Engineering for Cold Region & Heilongjiang Provincial Key Laboratory of Ecological Restoration and Resource Utilization for Cold Region & Key Laboratory of Microbiology, College of Heilongjiang Province & School of Life Sciences, Heilongjiang University, Harbin 150080, China; yaoweige0127@163.com (W.Y.); sunrui0823@126.com (R.S.); zhangwen11112020@163.com (W.Z.); kangjie182@126.com (J.K.); wzc130522@163.com (Z.W.); liangyangmao@126.com (L.M.); yangying010427@126.com (Y.Y.); lishuo228@163.com (S.L.); songgang@hlju.edu.cn (G.S.)

**Keywords:** *Lacticaseibacillus paracasei* HD1.7, acetate switch, quorum sensing, transcriptomic analysis, bioprocess

## Abstract

Acetate may act as a signaling molecule, regulating Paracin 1.7 production via quorum sensing (QS) in *Lacticaseibacillus paracasei* HD1.7. The “acetate switch” phenomenon requires mechanistic exploration to optimize Paracin 1.7 production. The “acetate switch” phenomenon delays with higher glucose levels (30 h, 36 h, and 96 h). Before the occurrence of the “acetate switch”, the ATP content increases and peaks at the “acetate switch” point and the NAD^+^/NADH ratio decreases, indicating energy changes. Moreover, the QS genes used for the pre-regulation of bacteriocin, such as *prcKR*, *comCDE*, were highly expressed. After the “acetate switch”, the ATP content decreased and the QS genes for the post-regulation of bacteriocin were highly expressed, such as *rggs234* and *sigma70-1*/*70-2*. The “acetate switch” could act as an energy switch, regulating bacterial growth and QS genes. Before and after the “acetate switch”, some metabolic pathways were significantly altered according to the transcriptomic analysis by HD1.7 and HD1.7-Δ*pta*. In this study, acetate was used as an input signal to regulate the two-component system, significantly influencing the bacteriocin expression system. And this study clarifies the roles of acetate, energy, and quorum sensing in promoting Paracin 1.7 production, providing a theoretical basis for optimizing the bacteriocin fermentation process of HD1.7.

## 1. Introduction

Quorum sensing (QS) is an intercellular communication mechanism among bacteria mediated by quorum signaling molecules (QSM). QS represents a cell-to-cell communication mechanism in bacteria, mediated through the synthesis, detection, and response to extracellular signaling molecules. This system enables bacterial populations to synchronize collective behaviors based on shifts in population density and vicinal community composition [1]. The phenomenon of QS was first discovered in the 1970s through studies of bioluminescence in *Vibrio fischeri* [2]. This groundbreaking finding revealed that bacteria engage in chemical communication by secreting signaling molecules into their environment. When the concentration of these signaling molecules reaches a critical threshold, bacteria can sense their population density and subsequently activate the expression of specific genes, thereby coordinating collective behaviors. Since this initial discovery, research on QS has advanced rapidly. It has now been conclusively demonstrated that QS plays pivotal roles in diverse biological processes, including antibiotic production, biofilm formation, virulence factor synthesis, and bioluminescence regulation [3]. The widely used QSM include autoinducing peptides, N-acyl-homoserine lactones (AHLs), and autoinducer-2 (AI-2) [4,5,6]. In 2003, Wolfe et al. demonstrated that acetyl phosphate, an intermediate of acetate metabolism, was involved in *Escherichia coli* biofilm formation as a global signal molecule [7]. Previous studies on *Lacticaseibacillus paracasei* HD1.7 reported a high expression of QS-related genes (QSRGs) *prcK*, *prcR*, *comR*, and *sigma* upon adding exogenous acetate. Also, the production of bacteriocin Paracin1.7 was improved. At the same time, some metabolic pathways such as histidine metabolism, pyrimidine metabolism, and amino acid biosynthesis were disturbed [8,9,10,11]. These results suggested that acetate and its related metabolites might act as signaling molecules involved in QS.

Acetate content is often under the “on-off” status due to carbon metabolism and energy variation in the cell. That is, the rapid growth of cells under aerobic conditions leads to the production of organic acids via overflow metabolism in bacteria, fungi, and higher organisms, such as acetate overflow in bacteria [12], Crabtree effect in yeast [13,14], and Warburg effect in mammals [15]. This is termed as “acetate on”. Among these processes, bacterial acetate overflow metabolism is considered to be an independent pathway beneficial to bacteria on the saturation of the enzymes in glycolysis and Krebs cycle. It can provide the cell with a potent tool to profit from ecological stress in population competition [16].

Acetyl-CoA can be oxidized to acetate with phosphotransacetylase and acetate kinase. High concentrations of acetate also serve as a back-up carbon source for conversion into acetyl-CoA, a process that helps to slow human aging [17,18]. Such a decrease in acetate concentration is “acetate off” [19]. During the conversion between acetate and acetyl-CoA, many intermediates become the basis for intracellular matter cycling and energy variation owing to the presence of acetyl and phosphate groups [20].

QS depends on cell density (matter cycle) and ATP consumption (energy flow), and the “acetate switch” is related to not only acetate content but also energy conversion in the cell. Hence, finding the cellular “acetate switch” points implies unlocking the secret of QS regulated by acetate overflow and energy metabolism.

Previous studies have shown that exogenously added acetate could regulate QS; however, the role of intracellular acetate remains unclear. The objectives of this study are as follows: to analyze the regulatory rules of glucose concentration on the “acetate switch” point in HD1.7, clarify the dynamic associations between acetate, energy metabolism, and quorum sensing before and after the “acetate switch”, and to provide a theoretical basis for optimizing the bioprocesses of bacteriocin production from the perspective of the “acetate switch”. Based on these objectives, the following aspects of exploration were conducted: (1) the effect of glucose concentration on the time point of the “acetate switch” of HD1.7; (2) the changes in growth, energy, and acetate metabolism of HD1.7 before and after the “acetate switch”, and the association between the “acetate switch” and QS; (3) the correlation between intracellular acetate content, bacterial physicochemical indicators, and acetate metabolism-related genes (AMRGs); and (4) the changes in differentially expressed genes and metabolic pathways in HD1.7 before and after the “acetate switch”. This study enriched our understanding of the role of the “acetate switch” mechanism in bacterial metabolic processes, defined the dual regulation of bacterial metabolism and community sensing by the “acetate switch”, and laid the foundation for further research on bacteriocins for promoting the industrialization of lactic acid bacteria.

## 2. Materials and Methods

### 2.1. Strain and Medium

HD1.7 (CCTCCM 205015) was provided by the Key Laboratory of Microbiology of Heilongjiang University (Harbin, China) [9,10,21]. The MRS medium required for the growth of HD1.7 included 10 g/L soy peptone, 0.2 g/L MgSO_4_·7H_2_O, 0.05 g/L MnSO_4_, 10 g/L beef extract, 5 g/L yeast extract, 20 g/L glucose, 2 g/L K_2_HPO_4_, 0.1 g/L Na_2_SO_3_, 5 g/L sodium acetate, 0.4 g/L ammonium citrate, and 1 mL Tween-80 (Polyoxyethylene sorbitan monooleate). MRS was adjusted to pH 5.5 and sterilized at 108 °C for 20 min. The reagents required for the above media were purchased from Beijing Auboxing Biotechnology, China. The influence of glucose concentration on the “acetate switch” points was investigated using 2, 5, and 20 g/L glucose in an MRS liquid medium. Three treatments were set up according to glucose concentration: Glu2 (2 g/L), Glu5 (5 g/L), and Glu20 (20 g/L), respectively.

### 2.2. Construction of the Pta-Deficient Strain of HD1.7

The acetate content of HD1.7 was reduced after “acetate switch”. Therefore, the *pta*-deficient strain was used in this study as a negative control. Both flanks of *pta* were amplified with primers *Eco*R I-*pta*-up-F, *Xba* I-*pta*-up-R, *Xba* I-*pta*-down-F, and *Sal* I-*pta*-down-R to harvest *pta*-up (500 bp) and *pta*-down (500 bp) fragments with polymerase chain reaction (PCR). A cloning vector pGEM-T-∆*pta* was constructed by ligating pre-digested pGEM-T, *pta*-up, and *pta*-down with *EcoR*I and *Xba*I. pKR6K is a conjugative plasmid, on which *sacB* can encode fructokinase to hydrolyze sucrose to fructan. When *sacB* is transferred to the recipient cell, it can cause cell death because fructan is toxic to the bacterium. The suicide plasmid pKR6K-∆*pta* was prepared after digesting pKR6K and pGEM-T-∆*pta* with *EcoR*I and *Sal*I and then ligating with T4 ligase.

Further, 0.5 mL of HD1.7 and 1 mL of ZW01 (strain obtained by introducing pKR6K-Δ*pta* into the recipient cell *E. coli* S17-1 λpir using heat shock) were centrifuged at 12,000 rpm for 2 min. The pellet was resuspended in 500 μL of 10 mM MgSO_4_, and the suspension was centrifuged again at 12,000 rpm for 2 min. The pellet thus obtained was resuspended in 20 μL of 10 mM MgSO_4_, dropped onto a solid MRS medium, and incubated at 37 °C for 24–48 h to facilitate conjugation between the donor and the receptor. The mixture was diluted 100-fold. Then, 100 μL of it was spread on an MRS solid medium containing 100 μg/mL Kan and further incubated for 16–20 h to finish homologous recombination. HD1.7-Δ*pta* could grow due to the presence of Kan, whereas the strain without the introduced plasmid did not survive (Figure S1).

### 2.3. Fermentation Parameters of HD1.7

The seed cultures of HD1.7 were inoculated into an MRS medium at a concentration of 2% (*v*/*v*) at 140 rpm and 37 °C for a certain time. For Glu2 and Glu5, the samples were taken at 1 h intervals for 12 h, followed by 18, 24, 30, 36, 48, 60, and 72 h. For Glu20, the samples were taken at 2 h intervals for 12 h, followed by 18, 24, 30, 36, 48, 60, 72, 84, 96, 108, and 120 h [10].

The cells in the collected fermentation broth were centrifuged at 8000 rpm and room temperature for 10 min to harvest the cell pellet. And then cell disruption was performed using an ultrasonic processor (SCIENTZ-IID, Ningbo Scientz Biotechnology Co., Ltd., Ningbo, China) at 200 W power with ice-bath cooling. The treatment consisted of 30 cycles of ultrasonication (3 s on/10 s off per cycle) to ensure complete cell lysis, thereby releasing intracellular components for the accurate analysis of membrane-barrier-isolated substances [22]. This procedure was repeated 30 times in an ice bath at 200 W. The intracellular pH was determined with an FE20 pH meter. High-performance liquid chromatography was employed to determine the acetate (intracellular and extracellular) and residual glucose contents [23,24,25]. The number of viable HD1.7 was measured by the plate colony counting method. The pyruvate content assay kit (Shanghai Jingkang Biotechnology Co., Ltd., Shanghai, China), coenzyme I NAD(H) content assay kit (Beijing Solarbio Science & Technology Co., Ltd., Beijing, China), and ATP content assay kit (Jiangsu Suke Biotechnology Co., Ltd., Nantong, China) were used to detect pyruvate content, NAD^+^/NADH ratio, and ATP content, respectively.

### 2.4. Determination of Gene Expression Levels Using qRT-PCR

RNA of HD1.7 was extracted using the total RNA extraction kit (Tiangen Biotech Co., Ltd., Tiangen, China), and its purity and concentration were determined using a Nanodrop 2000 spectrophotometer (Thermo Scientific Co., Ltd., Waltham, MA, USA). The first strand of cDNA was amplified by reverse transcription using the BioRT cDNA first-strand synthesis kit (TransGen Biotech Co., Ltd., Beijing, China). A real-time PCR system was established, including a cDNA template, primers, SYBR Mastermix (Vazyme Biotech Co., Ltd., Nanjing, China), and deionized water, to measure the expression of AMRGs. These AMRGs included acetate kinase genes (*ackA1* and *ackA2*), pyruvate oxidase genes (*poxB1*, *poxB2*, and *poxB3*), and phosphotransacetylase gene (*pta*). QS-related genes were also detected. These included competence-stimulating peptide genes (*comC*, *comD*, *comE*, and *comS*), AI-2 synthase gene (*luxS*), sensor kinase gene (*prcK*), response regulator gene (*prcR*), Rgg-family regulator genes (*rgg2*, *rgg3*, and *rgg4*), sigma factor genes (*sigma X*, *sigma70-1*, and *sigma70-2*), and bacteriocin gene. The values normalized by 16s rDNA were used to quantify relative gene expression by the 2^−ΔΔCT^ method [24]. The primer sequences used in the study are shown in Table S1.

### 2.5. Transcriptomic Analysis

The transcriptomic analysis was performed to verify the effect of the “acetate switch” on the gene expression and physiological activities of HD1.7 and HD1.7-Δ*pta*. Glu5 was fermented for 8 h (named as *a*) and 42 h (named as *b*), whereas HD1.7-Δ*pta* was cultured for 42 h (named as *c*). The three treatment samples were selected to perform transcriptomic analysis at Shanghai Majorbio Bio-Pharm Technology Co., Ltd. (Shanghai, China). The process was repeated three times for each sample. *b* and *a* were contrast before and after the “acetate switch”, whereas *b* and *c* had low acetate concentrations.

### 2.6. Statistical Analysis

The results were presented in the form of three parallel samples along with their standard deviations. Statistical and graphical analyses were conducted using Origin 2024 software (OriginLab Corp., Northampton, MA, USA). And the statistical significance was set as *p* < 0.05. All data were reported as means ± standard deviation (three biological replicates were set up) and analyzed by ANOVA (one-way ANOVA). OmicStudio online platform (https://www.omicstudio.cn/analysis, accessed on 23 December 2023), lingbo Microclass (http://cloud.biomicroclass.com/CloudPlatform/home, accessed on 25 December 2023), Oebiotech platform (https://cloud.oebiotech.com/#/home, accessed on 2 January 2024), and Bioinformatics platform (https://www.bioinformatics.com.cn/, accessed on 6 January 2024) were chosen to perform heat maps, gene-pathway enrichment network diagrams, volcano plots, correlation analysis, Venn diagrams, and WGCNA analysis. The false discovery rate (FDR) was used to determine the threshold for *p* values in multiple tests (Table S2). A threshold of FDR ≤ 0.05 and an absolute value of fold change (FC) ≥ 1.2 or FC ≤ 1/1.2 were used in the analysis as the criteria for determining the significance of differences in gene expression. Then, differentially expressed genes (DEGs) were subjected to the Kyoto Encyclopedia of Genes and Genomes (KEGG) enrichment analysis. Structural equation modeling (SEM) was performed using IBM SPSS AMOS (version 23.0).

## 3. Results and Discussion

### 3.1. Determination of the “Acetate Switch” Points Under Different Concentrations of Glucose

**Figure 1:**
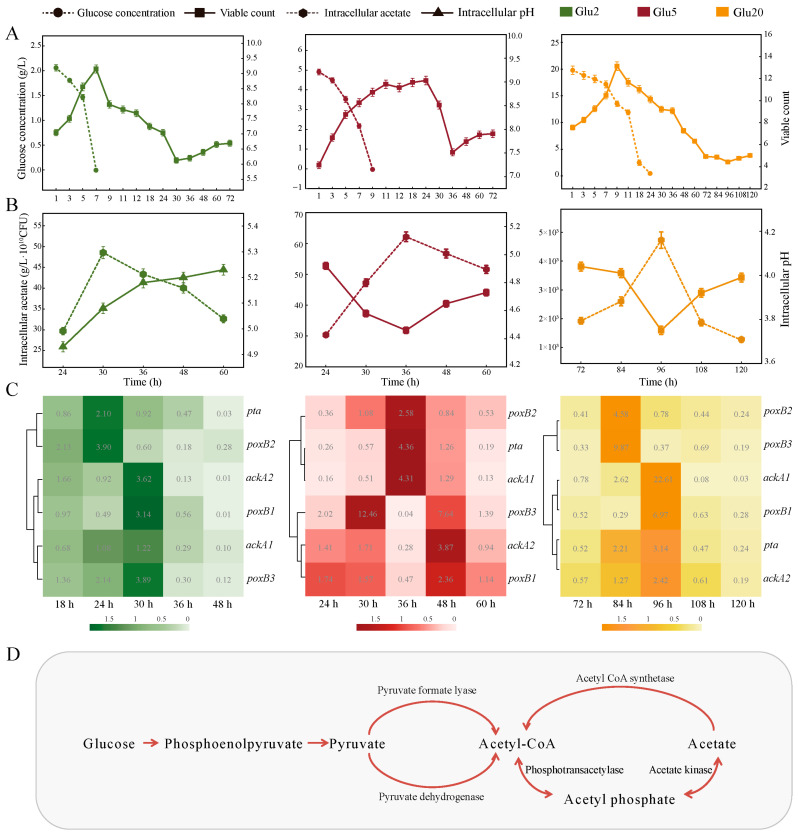
Determination of the “acetate switch” points under different concentration of glucose. (**A**) Changes in intracellular acetate content as well as intracellular pH in the three treatments (Glu2, Glu5, Glu20) as time progressed; (**B**) Glucose consumption as well as bacterial growth in the three treatments as time progressed; (**C**) Heat map of the expression of AMRGs in the three treatments as time progressed; (**D**) Metabolic pathway map utilizing glucose and acetate.

The intracellular acetate content under all the three treatments showed a trend of increase and then decrease (Figure 1A), with an obvious “acetate switch” characteristic. Therefore, the inflection point of acetate content was identified as the “acetate switch” point [26]. This phenomenon was not uncommon, some halophilic archaea, such as Halococcus saccharolyticus and Haloferax volcanii, underwent the “acetate switch” during their growth on glucose [27].

The “acetate switch” points occurred at 30, 36, and 96 h, respectively, in the Glu2, Glu5, and Glu20 groups (Figure 1A). The intracellular acetate content in all three groups reached its peak at these time points, with Glu20 displaying higher acetate levels than Glu5 and Glu2, and Glu5 exhibiting higher levels than Glu2. The “acetate switch” in HD1.7 was delayed and the intracellular acetate content inducing this phenomenon was improved with the increase in exogenous glucose concentration (Figure 1A). HD1.7 metabolized glucose to acetate, which could be used as a backup carbon source for its growth and metabolic activities under stress (Figure 1D) [28]. In this study, the glucose concentration decreased over the fermentation period and it was completely consumed before the “acetate switch” points, which were 7, 9, and 24 h, respectively (Figure 1B).

The number of viable HD1.7 in Glu2, Glu5, and Glu20 increased and then decreased (up to a maximum of 1.43 × 10^9^ ± 3.68 × 10^8^, 1.08 × 10^9^ ± 6.45 × 10^7^, and 1.39 × 10^13^ ± 1.18 × 10^12^), showing an increase again in 30, 36, and 96 h (6.35 × 10^5^ ± 3.81 × 10^4^, 1.54 × 10^6^ ± 9.24 × 10^4^, and 2.75 × 10^4^ ± 1.65 × 10^3^), respectively. This secondary growth proved the backup carbon source role of acetate (Figure 1B). The intracellular pH also increased to 5.23 ± 0.02 (60 h in Glu2), 4.72 ± 0.03 (60 h in Glu5), and 3.99 ± 0.02 (120 h in Glu20) with the depletion of intracellular acetate (Figure 1A).

The expression of AMRGs could further explain the reason and genetic basis of “acetate switch” points (Figure 1C). Also, *pta*, *ackA1*, *ackA2*, *poxB1*, *poxB2*, and *poxB3* in Glu2, Glu5, and Glu20 were more highly expressed before the “acetate switch”. They were highly expressed in the early stage of fermentation, reached the maximum expression level when the “acetate switch” occurred, and then decreased compared with the previous stage over time after the “acetate switch”. The fold change of these genes in Glu2 could be up to 5.24 ± 0.05, 5.17 ± 0.05, 28.38 ± 0.28, 5.44 ± 0.05, 9.66 ± 0.10, and 11.50 ± 0.11 (*p* < 0.05), respectively. *pta* had the largest fold change of 2.38 ± 0.05 in Glu5; a 100-fold change was found in *ackA1* in Glu20 (*p* < 0.05).

### 3.2. Energy Variation Before and After “Acetate Switch” Points

**Figure 2:**
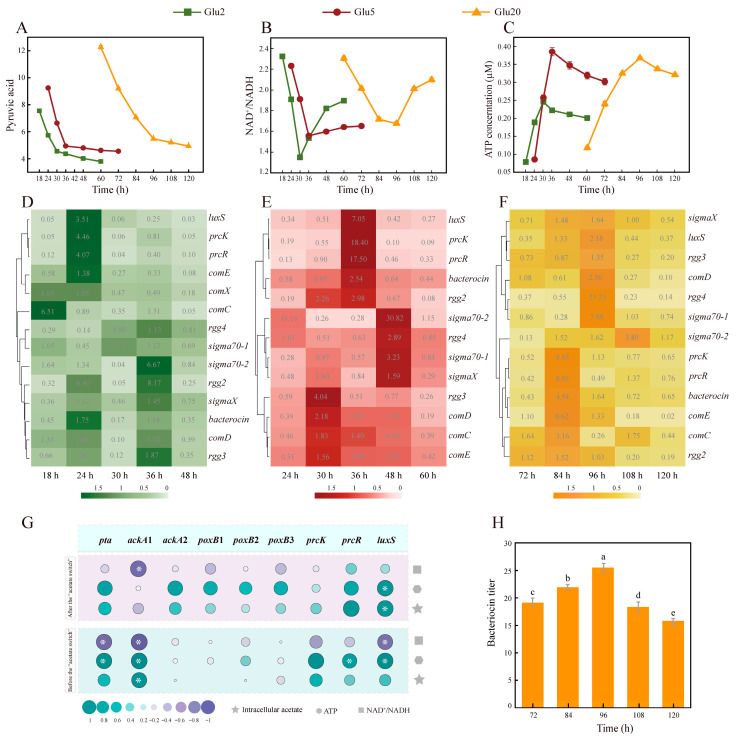
Variation in energy, AMRGs, and QSRGs abundance before and after “acetate switch” points. (**A**) Changes in pyruvate in the three treatments over time; (**B**) Changes in NAD^+^/NADH in the three treatments over time; (**C**) Changes in ATP in the three treatments over time; (**D**–**F**) Heat map of the expression of QSRGs in the three treatments over time; (**G**) Heat map of correlation analysis of QSRGs with acetate and energy before and after the “acetate switch”; (**H**) Bacteriocin titer of HD1.7 in Glu 20. “*” indicates “*p* < 0.05”; Different lowercase letters indicate significant differences among groups (*p* < 0.05).

Both the pyruvate content and NAD^+^/NADH ratio decreased (Figure 2A,B) before the “acetate switch” point, accompanied by ATP production in ATP, to 0.245 ± 0.003, 0.384 ± 0.01, and 0.366 ± 0.01 in Glu2, Glu5, and Glu20, respectively. This suggested glucose consumption by the bacteria to produce energy via pyruvate oxidation and electron transport (Figure 2C). However, the pyruvate utilization speed and ATP production decreased and the NAD^+^/NADH ratio increased after the “acetate switch” points (Figure 2A,B), resulting in lower energy production ability. The energy generation and intracellular acetate content were found to be consistent: before “acetate switch” points, the energy produced was conducive to cell growth and served as the energy pool for QS functionality. However, after “acetate switch” points, energy production was inhibited with the decline in cell growth (Figure 2C).

Pyruvate dehydrogenase (PDH) and pyruvate formate lyase could oxidate decarboxylated pyruvate to generate acetyl-CoA under aerobic and anaerobic conditions, which was converted into acetate via the *pta-ackA* operon [29,30]. This was a reversible low-affinity pathway, wherein the bacteria could also use acetate when its concentrations were high in the environment. This cycle resulted in a constant change in energy (Figure 1D).

### 3.3. Variation of QSRGs

QSRGs can all participate in QS to regulate bacteriocin synthesis and play different roles, for example, stress responses, bacteriocin production, biofilm formation, and virulence development, among others. The interspecies signaling molecule AI-2, autoinducing peptide Paracin 1.7, and acetate could all have been found to be able to regulate bacteriocin production using QS in HD1.7 [31,32,33]. In our previous research, the production of Paracin 1.7 was induced by pre-signal peptide CSP and the post-signal peptide XIP, respectively [31]. CSPs encoded by *comC* could regulate *prcK*, *prcR*, *luxS*, *comD*, and *comE* to promote bacteriocin gene expression, which is referred to as the pre-regulation system. The signaling peptide XIP encoded by *comS* activated the family of transcriptional regulators *rgg* (*rgg2*, *rgg3*, and *rgg4*), which could regulate stress responses, nutrient metabolism, bacteriocin production, biofilm formation, QS, and virulence formation [34,35]. *rgg* also regulated the expression of the global transcriptional regulator *sigma* (*sigmaX*, *sigma70-1*, and *sigma70-2*) to control biofilm formation and rapid adaptation of bacteria to global metabolic changes [24]. CSP-*prcK/R* could activate the expression of XIP-*rgg*-*sigma*, and hence XIP-*rgg*-*sigma* was also known as the post-regulation system.

Under all three treatments, QS genes (*prcK*, *prcR*, and *luxS*) and pre-regulation system genes, such as *comC*, *comD*, and *comE* were highly expressed before the “acetate switch” points, with the smallest fold change in *comC* expression (3.3 ± 0.03) and the largest one in *luxS* expression (9.48 ± 0.19) in Glu2 (*p* < 0.05) (Figure 2D). However, *prcK* (66 ± 1.32) and *comE* (29.3 ± 0.58) showed the largest fold changes in Glu5 and Glu20, respectively (*p* < 0.05) (Figure 2E,F). The expression of *ackA1* was significantly correlated with intracellular acetate and ATP levels and NAD^+^/NADH ratio before the “acetate switch” point. These correlations decreased after the “acetate switch”, suggesting the dependence of *ackA1* expression on intracellular acetate concentration. QS genes, such as *luxS*, were positively correlated with ATP both before and after the “acetate switch” point (*p* < 0.05) (Figure 2G), implying the involvement of acetate in intracellular energy flow as a common signaling molecule between species. Acetate can influence the expression levels of QS-related protein kinases in bacteria. These kinases can regulate downstream gene expression through phosphorylation processes. Additionally, the regulatory effect of acetate on protein kinase expression can enhance the stress response capability of bacteria to external environments. As observed in *Saccharomyces cerevisiae* SPSC01 fermentation studies, acetate can enhance cellular stress response capacity via the following mechanism: signaling molecules bind to the receptor PYR/PYL/RCAR, triggering phosphorylation to regulate the expression of genes such as *srk2e*/*ost1*/*snrk2.6*, thereby enhancing cellular stress response capacity [36].

Combined with the intracellular acetate content before the “acetate switch” points (Figure 1A), it was inferred that acetate might act as a signaling molecule to activate QS in HD1.7, resulting in high expression of signal transduction genes *prcK* and *prcR* and interspecific signaling molecule *luxS*, which was not only beneficial to the production of Paracin 1.7 but also consumed energy (Figure 2C). Our previous study showed that the exogenous addition of acetate could improve the concentration of Paracin 1.7 by 133.92%, which was accompanied by the high expression of *prcK* and *prcR* [9].

Around or after the “acetate switch” points, the post-regulation system genes, such as *sigmaX*, *sigma70-1*, *sigma70-2*, *rgg2*, *rgg3*, and *rgg4*, were highly expressed in Glu2, Glu5, and Glu20, with the highest fold change of 16.69 ± 0.33 (*p* < 0.05). After the “acetate switch” points, the extracellular acetate contents were 4.34 g/L (30 h in Glu2), 4.72 g/L (36 h in Glu5), and 5.80 g/L (96 h in Glu20) whereas the intracellular acetate content reached 48.55 g/L·10^10^ (30 h in Glu2), 62.16 g/L·10^10^ (36 h in Glu5), and 4700 g/L·10^10^ CFU (96 h in Glu20) [32].

Our research team previously found that low concentrations of acetate (2 g/L and 6 g/L) were more favorable to upregulate the pre-regulation system. In contrast, high concentrations of acetate (10 g/L) were inclined to upregulate the post-regulation system, which was consistent with the findings of this study [9]. In brief, intracellular acetate served as a switch for energy flow and gene expression, whereas extracellular acetate concentration helped to regulate gene expression.

Obviously, HD1.7 entered into the late stationary stage after the “acetate switch” points, characterized by the accumulation of various organic acids and secondary metabolites. The cells experienced stress under each treatment to increase the expression of *sigma70-1*, *sigma70-2*, and *sigmaX*. Moreover, the cell populations could also form biofilms to deal with such a situation; *rgg2*, *rgg3*, and *rgg4* were overexpressed. In fact, Meng et al. also found that acetate could enhance the resistance of *Lactobacillus plantarum*, *Lactobacillus sakei*, and *Lactobacillus rhamnosus* to *Staphylococcus aureus* [37].

### 3.4. SEM Analysis

**Figure 3:**
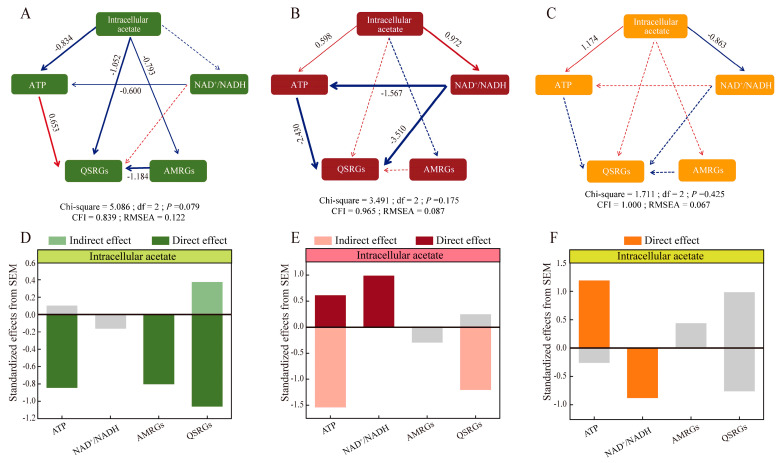
SEM analysis of the effects of intracellular acetate on energy as well as quorum sensing. (**A**,**D**) SEM analysis of Glu2, direct and indirect effects of *Lacticaseibacillus paracasei* HD1.7 intracellular acetate on ATP, reducing power, AMRGs, and QSRGs. (**B**,**E**) SEM analysis of Glu5, direct and indirect effects of *Lacticaseibacillus paracasei* HD1.7 intracellular acetate on ATP, reducing power, AMRGs, and QSRGs. (**C**,**F**) SEM analysis of Glu20, direct and indirect effects of *Lacticaseibacillus paracasei* HD1.7 intracellular acetate on ATP, reducing power, AMRGs, and QSRGs. Differently colored bars represent different groups; gray bars indicate statistically tested *p* > 0.05. Red symbolizes a positive correlation, and blue symbolizes a negative correlation; solid lines indicate a significant effect between variables, while dashed lines indicate a non-significant effect.

Acetate (intracellular/extracellular) concentrations were more directly related to energy. The correlation between intracellular acetate and ATP changed from −0.834 (*p* < 0.001) (Glu2) to 0.598 (*p* < 0.001) (Glu5) and 1.174 (*p* < 0.001) (Glu20) upon adding glucose. A similar situation occurred in the relationship between intracellular acetate and NAD^+^/NADH (Figure 3). In Glu2, low concentrations of acetate (intracellular/extracellular) directly influenced both QSRGs and AMRGs with correlation coefficients of −1.052 (*p* < 0.001) and −0.793 (*p* < 0.001), and also indirectly affected QSRGs and AMRGs via ATP (Figure 3A,D).

In Glu5, acetate (intracellular/extracellular) did not directly influence QSRGs, but indirectly influenced QSRGs via ATP and NAD^+^/NADH (−2.430 and −3.510, *p* < 0.001); energy served as an intermediate bridge (Figure 3B,E).

However, in Glu20, the effects of ATP and NAD^+^/NADH on QSRGs and AMRGs were not significant, probably due to the high concentration of acetate (intracellular/extracellular) (Figure 3C,F).

### 3.5. Changes of Genes in HD1.7 with Different Acetate-Producing Capacity Before and After the “Acetate Switch”

**Figure 4:**
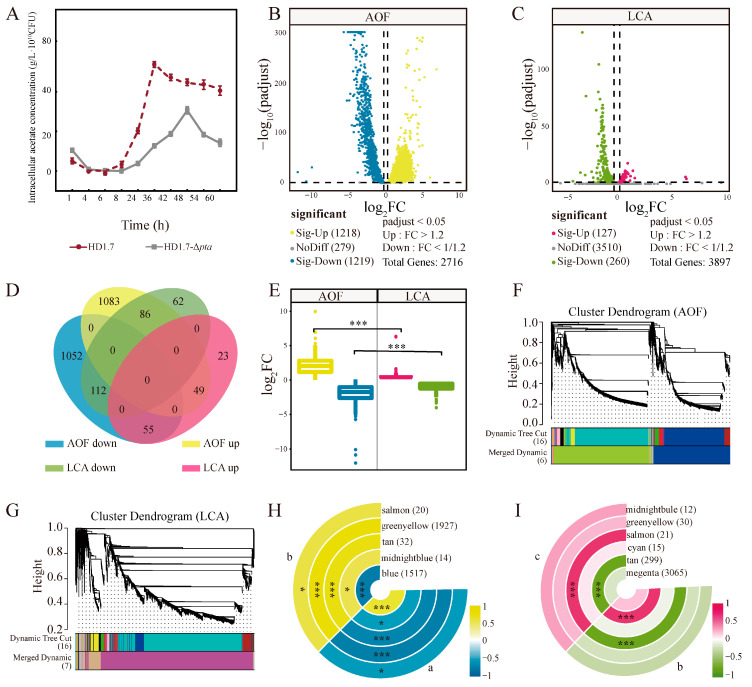
Changes of genes in HD1.7 with different acetate-producing capacity before and after the “acetate switch”. (**A**) Intracellular acetate content of HD1.7 and strain HD1.7-Δ*pta* under 5 g/L glucose conditions; (**B**) Volcano gram of DEGs in the AOF group; (**C**) Volcano gram of DEGs in the LCA group; (**D**) Venn diagram of DEGs in AOF vs. LCA; (**E**) Variance analysis of DEG in LCA and LCA, the color labeling involved in this figure is consistent with that in 4D.; (**F**) WGCNA of DEGs in the AOF group; (**G**) WGCNA of DEGs in the LCA group; (**H**) Ringed heat map of modular trait in WGCNA associations of AOF, where each ring represents a module, the letters in the figure indicate the treatment groups; (**I**) Ring heatmap of module trait associations in WGCNA of LCA, where each ring represents a module, the letters in the figure indicate the treatment groups. “*” indicates “*p* < 0.05”; “***” indicates “*p* < 0.001”.

Under the same fermentation conditions, the intracellular acetate content of strain HD1.7-Δ*pta* was lower than that of the normal strain HD1.7 (Figure 4A). The visual analysis using volcano plots revealed that 1218 and 127 DEGs were significantly upregulated in the *b* vs *a* (samples were before and after “acetate switch”, AOF) and *c* vs *b* (samples all had a low content of acetate, LCA) (*p* < 0.05), respectively, whereas 1219 and 260 DEGs were significantly downregulated in the two groups (Figure 4B,C)

Venn diagram analysis showed 49 and 112 commonly significantly upregulated and downregulated DEGs, respectively, among the DEGs (Figure 4D). The number of more significant DEGs was higher in the AOF than in the LCA, which might be due to the more obvious changes before and after the “acetate switch” in the same strain. Significant differences were found between up- and downregulated genes in both groups (Figure 4E) (*p* < 0.001).

WGCNA analysis was performed to understand the functional module classification of the DEGs (all DEGs with expression levels lower than 1 were deleted, the threshold for module merging was 0.25, and the minimum number of genes in the module was 10). Five functional modules (blue, midnight blue, tan, yellow-green, and light pink) in AOF were clustered, each comprising 1517, 14, 32, 1927, and 20 DEGs, respectively. Moreover, 3065, 299, 15, 21, 30, and 12 DEGs in magenta, sepia, cyan, light pink, yellow-green, and midnight blue modules, respectively, were classified in LCA (Figure 4F,G). Blue, midnight blue, tan, yellowish green, and light pink functional modules were significantly correlated with the AOF. In contrast, LCA had a strong relationship with dark brown and light pink functional modules (Figure 4H,I).

### 3.6. Level in Metabolic Pathways of HD1.7 with Different Acetate-Producing Capacity Before and After the “Acetate Switch”

#### 3.6.1. KEGG Enrichment

**Figure 5:**
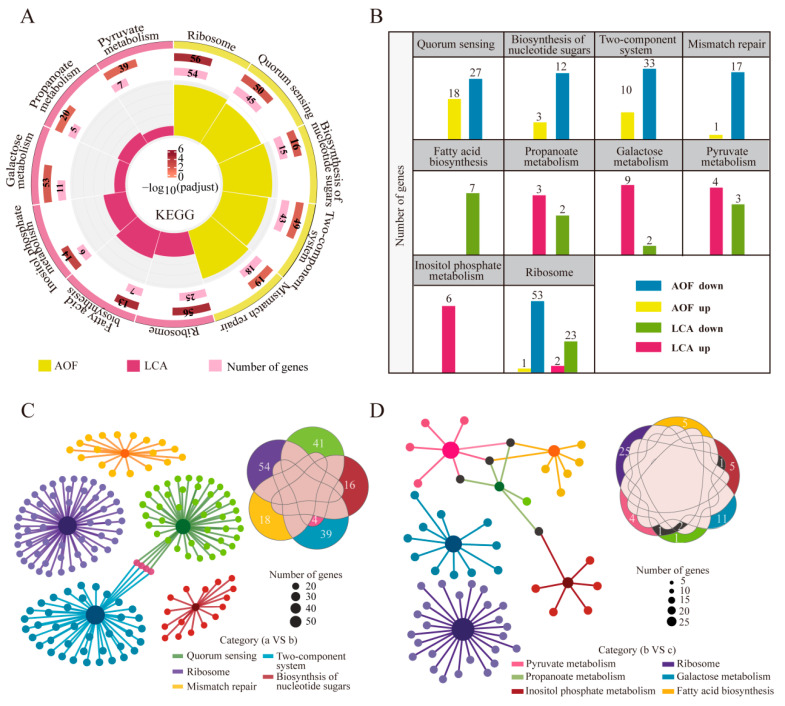
Changes in metabolic pathways of HD1.7 with different acetate-producing capacity before and after the “acetate switch”. (**A**) KEGG enrichment in AOF and LCA to analyze differential metabolic pathways and their enriched genes; (**B**) histogram of the number of up- and downregulated genes in the differential metabolic pathways of AOF and LCA; (**C**) dynamic Venn diagram of enriched genes in the differential metabolic pathways of AOF; (**D**) dynamic Venn diagram of enriched genes in the differential metabolic pathways of LCA.

Five metabolic pathways were found to be significantly enriched in AOF (*p* < 0.05), namely, ribosomal pathway, QS, biosynthesis of nucleotide sugars, two-component system, and mismatch repair pathway (Figure 5A). Each of these metabolic pathways influenced a wide range of life activities in HD1.7. The ribosomal pathway, biosynthesis of nucleotide sugars, and mismatch repair pathway were all related to the most basic bacterial DNA replication, transcription, and translation; all of these impacted bacterial growth, metabolism, and stress responses [38]. These metabolic pathways changed significantly before and after the “acetate switch” (*p* < 0.05), indicating the importance of “acetate switch” points. The expression of QS and the two-component system further confirmed the close relationship between “acetate switch” points and these systems.

In the LCA, the ribosomal pathway, pyruvate metabolism, propionate metabolism, galactose metabolism, inositol phosphate metabolism, and fatty acid biosynthesis pathway were significantly enriched (*p* < 0.05), which were all related to acetate metabolism (Figure 5A). Both b and c groups had low acetate content, due to the consumption of acetate as the backup carbon source when HD1.7 grew to the late stationary stage in the former case, and the change in acetate metabolism in the latter case. This also explained the cellular response to acetate metabolism before and after the “acetate switch” point. Overall, both AOF and LCA were co-enriched in the ribosome pathway, with 54 and 25 DEGs, respectively, suggesting that this pathway was jointly influenced by acetate content. The number of downregulated genes in each pathway was higher in AOF, whereas the number of upregulated genes in each pathway was higher in LCA, except for the inositol phosphate metabolism (Figure 5B).

In AOF, four genes, *ciaH*, *ciaR*, *nisK*, and *agrA*, were shared between the QS system and the two-component system. During the “acetate switch” took place, bacteria might respond to stress via QS (Figure 5C). In LCA, *accB*, *pflB*, and *accC*, which are involved in propionate metabolism and pyruvate metabolism, were chosen. The “acetate switch” modified the central carbon metabolism of the bacteria, aiming to enhance the bacterial resilience and their ability to adapt to environmental changes (Figure 5D).

#### 3.6.2. QS and Two-Component System

**Figure 6:**
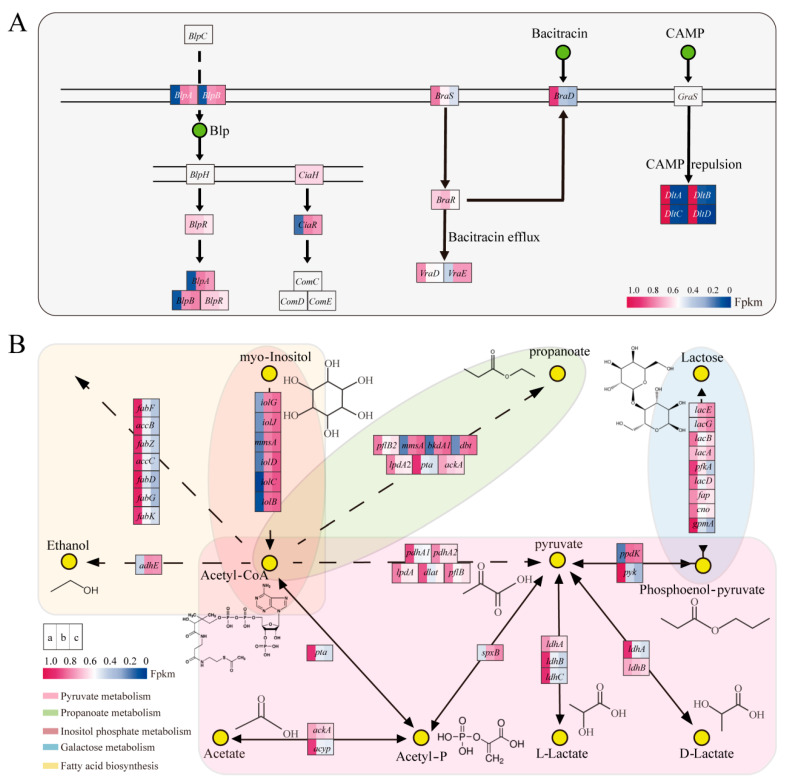
Analysis of differential metabolic pathways in AOF and LCA. (**A**) QS and two-component system; (**B**) The other metabolic pathway. Solid arrows represent a direct reaction from the previous substance to the next, dotted arrows indicate that the reaction requires a complex process. Created with BioRender.com.

**Figure 7:**
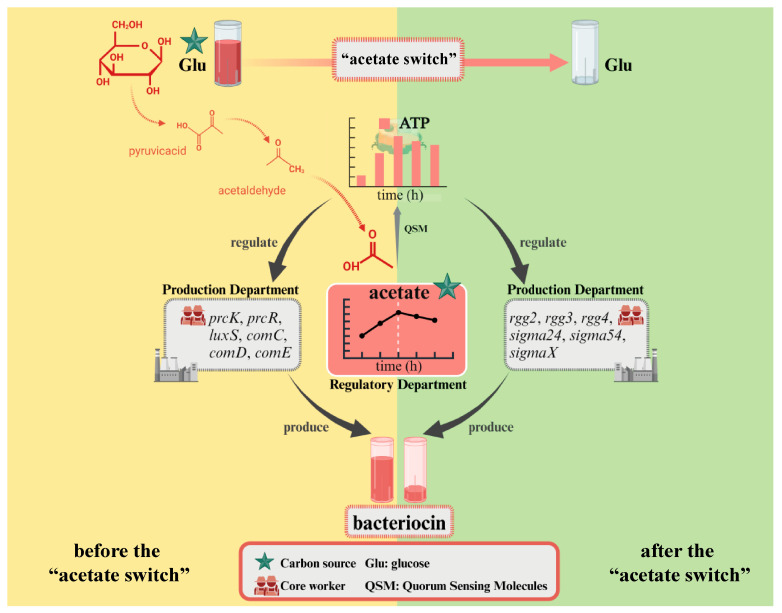
Conceptual model of production of bacteriocin by *L. paracasei* before and after the “acetate switch”. Created with BioRender.com.

*ComC*, *comD*, and *comE* belonged to the pre-regulation system and influenced the production of Paracin 1.7, their phosphorylated protein gene, *ciaR*, was upregulated in AOF, indicating that the production of bacteriocins increased with the progression of fermentation (Figure 6A and Figure 7). *CiaR* belongs to the phosphorylated protein genes and promotes bacteriocin production through a phosphorylation process. *ciaR* was also associated with biofilm formation, contributing to bacterial survival and competition [39]. Also, its expression was downregulated in LCA (Figure 6A), suggesting the engagement of cells in resistance to stressful environments by producing biofilms when the acetate metabolic pathway was blocked.

*BlpA* and *blpB* are ABC transporter protein genes, which can export QS signaling molecules, an important component involved in QS [40]. These proteins serve as critical components of the QS system, facilitating communication between bacteria. These two genes were upregulated in AOF and downregulated in LCA. This finding was consistent with the results of Kang et al., who found that either exogenous acetate addition or internal acetate content adjustment influenced ABC transporter proteins [9]. As ABC transporters were the stabilizers of *comC*, *comD*, and *comE*, the high expression of *blpA* and *blpB* contributed to the production of Paracin 1.7.

*DltA*, *dltB*, *dltC*, and *dltD* are two-component system genes that can modify cell wall composition and antibiotic resistance ability in Gram-positive bacteria [41]. Moreover, *braD*, *braS*, *braR*, and *vraD* are linked to bacitracin production [42]. *DltA*, *dltB*, *dltC*, *dltD*, *braD*, *braS*, *braR*, and *vraD* were downregulated in the AOF and LCA with the progression of fermentation. The resistance ability changed in the later growth stages or under the blocked acetate metabolic pathway.

#### 3.6.3. The Other Metabolic Pathway

The expression levels of *accB*, *accC*, *fabD*, *fabF*, *fabG*, *fabK*, and *fabZ* were lower in AOF and LCA. They are involved in the synthesis of long-chain fatty acids and affect cellular resistance through cell membrane fluidity, as well as QS [43] (Figure 6B). Fatty acid metabolism was influenced by salinity, temperature, pH, and oxidative stress [44], in addition to intracellular acetate content [45,46], which was essential for bacterial stress and adaptation [47].

Similarly, not only *pta*, *ackA*, and *acy*, but also central carbon metabolism genes (*ppdk*, *pyk*, *pdhA1*, *pdhA2*, *lpdA1*, *dlat*, and *pflB*), galactose metabolism pathway genes (*lacA*, *lacB*, *lacD*, *pfkA*, *fbp*, *eno*, and *gpmA*), and propanoate metabolism pathway genes (*pflB*, *lpdA*) were downregulated with the change in acetate metabolism.

## 4. Conclusions

The higher the glucose concentration, the longer the “acetate switch” point (30 h in Glu2, 36 h in Glu5, and 96 h in Glu20), indicating that the “acetate switch” was related to the concentration of added glucose under the glucose concentration gradient set in this experiment. The viable count of HD1.7 initially increased, then decreased, and subsequently displayed secondary growth, suggesting that intracellular acetate could be used as a backup carbon source to induce the secondary growth of bacteria. Energy increased before the “acetate switch” and decreased afterward. The “acetate switch” could act as an energy switch, regulating bacterial growth and QS before the “acetate switch” and affecting the production of Paracin 1.7 due to energy loss after the “acetate switch”. The discussion of the results revealed that acetate and energy jointly influenced Paracin 1.7 production through different divisions of labor (Figure 7). Moreover, the impact of the “acetate switch” on metabolic pathways such as fatty acid synthesis and QS further highlighted its importance in optimizing bacteriocin production. This study laid a theoretical foundation for improving the bacteriocin yield of probiotics in industrial production in the future.

## Data Availability

The original contributions presented in the study are included in the article/Supplementary Materials, further inquiries can be directed to the corresponding author.

## Supplementary Materials

The following supporting information can be downloaded at: https://www.mdpi.com/article/10.3390/foods14152691/s1, Figure S1: M: DNA Marker DL10000, Lane 1 is HD1.7-Δ*pta*, Lane 2 is wild type, and Lane 3 is control; Table S1: Primers sequences in the experiment; Table S2: The abbreviations mentioned in this study.
