# Supplementary material for: Optimizing the Bioprocesses of Bacteriocin Production in Lacticaseibacillus paracasei HD1.7 by the “Acetate Switch”: Novel Insights into the Labor Division Between Energy Metabolism, Quorum Sensing, and Acetate
Source: Foods. 2025 Jul 30;14(15):2691. doi: 10.3390/foods14152691 (PMC12346395; doi:10.3390/foods14152691)
Supplement: Supplementary file 1 [file foods-14-02691-s001.zip › foods-3756092-supplementary.pdf]

Supplementary Material

Optimizing the bioprocesses of bacteriocin production in *Lacticaseibacillus paracasei* HD1.7 by the "acetate switch": Novel insights into the labor division between energy metabolism, quorum sensing, and acetate

Supplementary Table S1. Primers sequences in the experiment

Serial number	Name		Sequence（5'→3'）	Length（bp）	purposes
1	*comC*	F	ACGCTTCGTGCGGAGAA	82	
		R	GCTTGGAAAAGATTGCTGGT		
2	*comD*	F	ATCCACACCGAAGGAACAA	175	
		R	CGTTGACGACGCTTGCTT		
3	*comE*	F	ATCGCTCCAATACCCAACA	191	
		R	TGGTAGCAGACTGTGGTTTATT		
4	*rgg1*	F	TGCGTCAGTTTGTAGACAGTTCA	210	
		R	GTTCACCTTTGCGGCATTTA		
5	*Rgg2*	F	ACCGAATCAGCAGAGCAAC	148	
		R	GGCAATACAATGACCCAGC		
6	*Rgg3*	F	GCTTTGTCGTTGGTTACTGTCA	158	
		R	AGTCTGGCAGTTGTCAGGTCTT		
7	*Rgg4*	F	CCTCAAAAAGGAGCAGGAATG	281	
		R	GAATCGTGGCTGTCGGTAAG		qRT-PCR
8	*Rgg5*	F	CTGGTCGCAATGGTTAGCA	227	
		R	AAGCACTGGGGACTTTATGAG		
9	*sigma24*	F	GGCTATTTTGACGCCTATGG	99	
		R	CGTTGCCGACTAATCTGTAATC		
10	*sigma54*	F	GATCTGAGTGTAGGATTGGCTATC	200	
		R	GAGAAACCGATAAACGCATAGT		
11	*sigma70-1*	F	GCAAAGTGTTTCCGCAAGA	191	
		R	CCGCTAGTCCTAACGGTGTT		
12	*sigma70-2*	F	TCAGGTTGACCGATGAGGA	134	
		R	GTCGTTGAGTTCGCCATAAT		
13	*sigmaX*	F	GCCAAGATGTGAATCGTCC	257	
		R	TTCGGCAGGCTATTACCC		
14	*bacteriocin*	F	CAGCTAGACTCCAGCCACTT	115	

		R	GGCGGTTTTGCATTTGT	
15	*competence1*	F	CGAATATCTGGACTCCTTTGTT	131
		R	CAAACGCTGCCACTGATAAC	
16	*competence2*	F	GCCAATGACGCCTAATAATCA	246
		R	TCGCCAATGCCAAAGAAAC	
17	*competence3*	F	ATGCTCGGCTGGATACCTT	267
		R	AATCGGCGTTTGTCTTGG	
18	*pta*	F	TGGTTGCGTCCTTTGTTG	183
		R	CCATACCGTCTGCTTCCTTC	
19	*ackA1*	F	CAACCCTGAAGTGGAAGC	146
		R	CAACCAACCCTAACGCCT	
20	*poxB1*	F	CCAGCAAAAGGCACTATC	115
		R	TCATCATCACGCAAGGAG	
21	*16s rRNA*	F	AGAAGAAGCACCGGCTAACTC	213
		R	CTCTACGCATTTCACCGCTAC	

Supplementary Table S2. The abbreviations mentioned in this study

**Abbreviation**	**Full Name**
AHLs	N-acyl-homoserine lactones
AI-2	autoinducer-2
AMRGs	acetate metabolism-related genes
DEGs	differentially expressed genes
FC	fold change
FDR	false discovery rate
Glu2	2 g/L
Glu20	20 g/L
Glu5	5 g/L
KEGG	Kyoto Encyclopedia of Genes and Genomes
L. paracasei	*Lacticaseibacillus paracasei*
PCR	polymerase chain reaction
PDH	Pyruvate dehydrogenase
QS	quorum sensing
QSM	signaling molecules

QSRGs	QS-related genes
SEM	Structural equation modeling
SEM	Structural equation modeling
Tween-80	Polyoxyethylene sorbitan monooleate

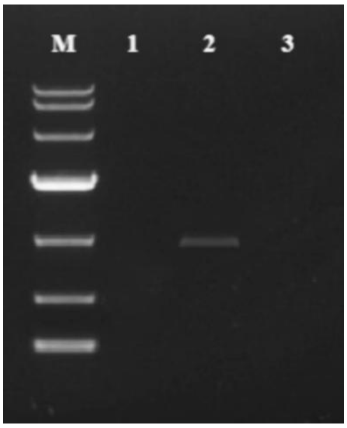

Supplementary Figure S1. M: DNA Marker DL10000, Lane 1 is HD1.7-*Δpta*, Lane 2 is wild type, and Lane 3 is control.
